# Performance evaluation of air-source heat pump based on a pressure drop embedded model

**DOI:** 10.1016/j.heliyon.2024.e24634

**Published:** 2024-02-09

**Authors:** Tim Koopman, Tingting Zhu, Wilko Rohlfs

**Affiliations:** Department of Thermal and Fluid Engineering, Faculty of Engineering Technology (ET), University of Twente, 7522, NB, Enschede, the Netherlands

**Keywords:** Air-source heat pump, Coefficient of performance, Pressure drop, Logarithmic mean temperature difference, Algorithm optimization

## Abstract

An air-source heat pump simulation model, accounting for evaporator and condenser pressure drop, has been developed. The model is capable of computing the heat pump's coefficient of performance (COP) under different ambient temperatures and relative humidities above frosting conditions. This research extends an existing iterative simulation method that relies on the equalization of logarithmic mean temperature differences (LMTDs) calculated through two different approaches by adding a pressure drop simulation. Frictional and acceleration pressure drop is considered, computed iteratively. Simulation results for three different refrigerants, R410A, R32 and R290, are compared. The model's accuracy is validated by comparing simulated COP values with measured COP values from the reference heat pump datasheet. The model closely replicates the measured COP values above frosting conditions, with only a slight underestimation of approximately 1.5%. Results show a substantial impact of ambient temperature on the COP. For instance, an ambient temperature of 20 ^*◦*^C, compared to 7 ^*◦*^C, results in a COP increase of up to 35%, while an ambient temperature of −10 ^*◦*^C leads to a 26% reduction in COP. Relative humidity enhances the COP if air moisture condensation becomes possible. Higher condenser capacities negatively affect the COP. The study highlights the differences in pressure drop characteristics between the condenser and the evaporator for the modeled heat pump, with maximum pressure drops of 220 kPa and 50 kPa for the condenser and evaporator, respectively. Additionally, the choice of refrigerant significantly influences pressure drop, with R32 displaying the lowest pressure drop, R410A showing the highest condenser pressure drop, and R290 causing the highest evaporator pressure drop.

## Highlights

• An air source heat pump model including the model of evaporator and condenser pressure drop is developed.
• The iterative simulation approach is based on the equalization of LMTD computed using two different methods.
• The developed model is well validated based on the reference data source.
• The effects of R-32, R-410A and R-290 refrigerants on pressure drop and performance have been evaluated.

## Nomenclature

**Symbols**
A: area, $m^{2}$
b: corrugation depth, m
Bd: Bond number, -
COP: coefficient of performance, -
D: diameter, m
f: Darcy friction factor, -
G: mass flux, $\text{kg}/\left(m^{2}\;s\right)$
g: gravitational acceleration, $m/s^{2}$
h: specific enthalpy, J/kg
K: two-phase number, -
L: length, m
LR: load ratio, -
$\dot{m}$: mass flow rate, kg/s
P: pressure, Pa
$\dot{Q}$: heat, W
Re: Reynoldsnumber,−
T: Temperature,°C
U: overall heat transfer coefficient, W/(m^2^ K)
v: specific volume, $m^{3}/\text{kg}$
$\dot{W}$: work, W
x: vapor quality, -

**Greek symbols**
γ: dimensionless corrugation parameter, -
Δ: difference
η: efficiency, -
$\dot{\lambda }$: corrugation wavelength, m
μ: dynamic viscosity, Pa s
φ: area enlargement factor, -
ρ: density, $\text{kg}/m^{3}$
σ: surface tension, N/m

**Subscripts**
1: heat pump cycle compressor inlet
2: heat pump cycle condenser inlet
3: heat pump cycle expansion valve inlet
4: heat pump cycle evaporator inlet
a: air
acc: acceleration
c: condenser or cross-sectional
com: compressor
dec: deceleration
e: evaporator
el: electricity
eq: equivalent
fan: fan
full Load: full load
*h*: hydraulic
i: inlet
$i^{\prime }$: virtual inlet
l: liquid
LM: logarithmic mean
lv: vapor-liquid difference
m: mean
n: new
o: outlet
$o^{\prime }$: virtual outlet
out: output
pc: percentage change
r: refrigerant or reference
s: isentropic or surface
satLiq: saturated liquid
satVap: saturated vapor
sub: subcooled
sup: superheated
t: total
tp: two-phase
v: vapor

**Abbreviations**
COP: coefficient of performance
CW: constant work
GWP: global warming potential
HFC: hydrofluorocarbon
LMTD: logarithmic mean temperature difference
ODP: ozone depletion potential
PHE: plate heat exchanger
RH: relative humidity

## 1 Introduction

Heating causes 50% of the global energy consumption and is responsible for 40% of global carbon dioxide emissions [1]. Within the EU, buildings play a pivotal role, representing 40% of the EU's energy consumption and contributing to 36% of the total greenhouse gas emissions [2]. A substantial 77% of heating within the EU is still based on fossil fuels [3]. Heating could become less polluting by increasing the share of heat pumps. A heat pump is a device designed to transfers heat from a cold to a hot reservoir through mechanical work input. It operates based on the thermodynamic principles of the refrigeration cycle. The necessary work input is generated by a compressor, which consumes electricity. The working fluid, a refrigerant, undergoes phase, temperature and pressure changes as it moves through the system. Many active heat pumps today typically use hydrofluorocarbons (HFCs), like R410A or R32, as refrigerants. However, HFCs are associated with a significant global warming potential (GWP) [4], prompting ongoing efforts in the EU to phase them out [5]. Consequently, there is a growing focus on alternative refrigerants with lower GWPs, such as the natural refrigerant R290, which however has the challenge of flammability [6]. The main advantage of heat pumps compared to other heating technologies is their efficiency. For each Watt of electrical input, a heat pump generates multiple Watts of thermal output, making it significantly more efficient than a resistance heater, which produces one Watt of heat per Watt of electrical input. Despite fossil fuels still constituting 36% of the electricity production in the EU [7], heat pumps have an environmental advantage over non-renewable heating sources such as gas boilers, since these rely exclusively on fossil energy. In the foreseeable future, there is an expected reduction in the use of fossil fuels for electricity generation within the EU, which will further enhance the environmental advantage of heat pumps [8]. In light of the environmental advantages compared to conventional fossil fuel technology and the heightened efficiency relative to resistance heaters, heat pumps emerge as a promising technology for the future.

The coefficient of performance (COP) of a an air-source heat pump is influenced by ambient temperature, to a lesser degree by relative humidity, and condenser-side conditions. The COP increases at higher ambient temperatures and relative humidities, while a higher condenser-side temperature decreases the COP. However, determining the exact sensitivity of these factors on the COP is not straightforward. Conducting experiments to ascertain this sensitivity is both time-consuming and expensive. Heat pump manufacturers typically provide the COP for only a few reference conditions. Heat pump simulation models are a solution to quickly determine the COP across a wide range of environmental conditions, and thereby cumbersome experiments can be avoided. Several studies have developed simulation models for various heat pump types to calculate the COP under a wide range of operating conditions. For instance, Kinab et al. [9] model each component of an air-to-water heat pump in detail and compute the COP. Evaporator and condenser are modeled using heat transfer correlations, while the compressor is modeled via curve fitting u sing manufacturer data. While t his approach yields a detailed model, it necessitates numerous input parameters that can be challenging to obtain. Moreover, the model does not explicitly account for relative humidity, and it simplifies the condenser and evaporator as isobaric. This assumption reduces the accuracy of the model, because real-world condensers and evaporators always suffer from pressure drop. Another model developed by Ibrahim et al. [10] adopts a moving-boundary approach to model the evaporator and condenser. The moving-boundary approach involves a system of partial differential equations, making it complex. Relative humidity is again not explicitly addressed and pressure drop is neglected.

Other studies model special types of heat pumps. Li & Wang [11] model an air-source heat pump featuring an additional solar and photovoltaic evaporator using heat transfer correlations. Such heat pump types are relatively uncommon. A study that addresses relative humidity is the work by Sezen & Gungor [12]. Using energy balances and the equalization of logarithmic mean temperature differences computed in two different manners in condenser and evaporator, an air-to-air heat pump is simulated and the COP change compared to a reference state for various ambient temperatures and relative humidities is analyzed above frosting conditions. Sezen & Gungor [12] also consider the evaporator and condenser to be isobaric. Other studies model pressure drop in evaporator and condenser, but put little emphasis on varying environmental conditions. For instance, Sarkar et al. [13] simulate a transcritical CO_2_ heat pump dryer and model pressure drop using single-phase and two-phase correlations, but their simplifying assumption is that ambient conditions remain constant. Miyara et al. [14] simulate a heat pump with different refrigerant mixtures, but pressure drop is only considered for the evaporator and not for the condenser. Additionally, ambient conditions are not varied. In another study, Han et al. [15] create a simulation model of a heat pump with waste heat recovery based on an experimental setup. Single-phase and two-phase correlations are employed to obtain the pressure drop, and the COP under different ambient temperatures is analyzed. However, relative humidity is not explicitly considered, and only a single refrigerant is regarded.

Our study introduces a novel approach that combines an analysis of the impact of ambient temperature and relative humidity on the COP with a pressure drop simulation. Building upon the simple and accurate simulation method of Sezen & Gungor [12], we enhance the accuracy of the COP calculations by introducing evaporator and condenser pressure drop. The following novelties are provided:

• Evaporator and condenser pressure drop is introduced, iteratively computed using single-phase and two-phase pressure drop correlations.
• A comparative analysis of COP values with and without pressure drop for different ambient conditions.
• Investigation of pressure drop with three distinct refrigerants: R410A, R32, and R290.
• Two calculation of logarithmic mean temperature difference (LMTD) computation methods ensures the accuracy of the final LMTD value.
• Enhanced efficiency in MATLAB calculations is achieved through the implementation of a parallelized solving procedure, accomplished by introducing the ‘fgoalattain’ function.

The paper proceeds with an explanation of the modeling approach in Section 2, including system setup, model assumptions, model options, the solving procedure, thermodynamic equations, two methods for computing LMTD, and evaporator and condenser pressure drop computations. Section 3 presents results for a reference heat pump and discusses the underlying reasons for these outcomes. Section 4 summarizes the key findings and outlines ideas for further research.

## 2 Methodology

### 2.1 Setup and assumptions

**Fig. 1:**
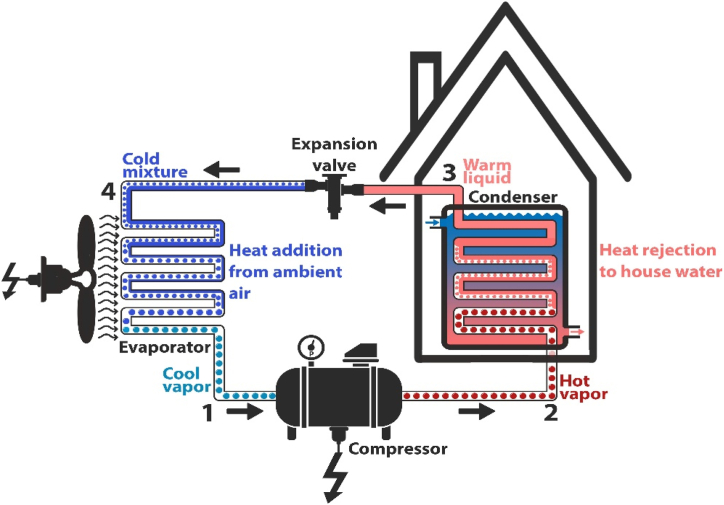
Set-up of an air-to-water heat pump.

An air-to-water heat pump comprises four key components: a compressor, a condenser, an expansion valve, and an evaporator, as depicted in Fig. 1. The evaporator draws heat from the air, while the condenser releases heat into water.

Five assumptions are applied:

1) The compressor has a constant isentropic efficiency of 80%.
2) The refrigerant in the evaporator is superheated 5 °C.
3) The refrigerant in the condenser is subcooled 5 °C.
4) Expansion through the expansion valve is regarded as isenthalpic.
5) There are no undesired heat losses in the compressor and the expansion valve.

**Fig. 2:**
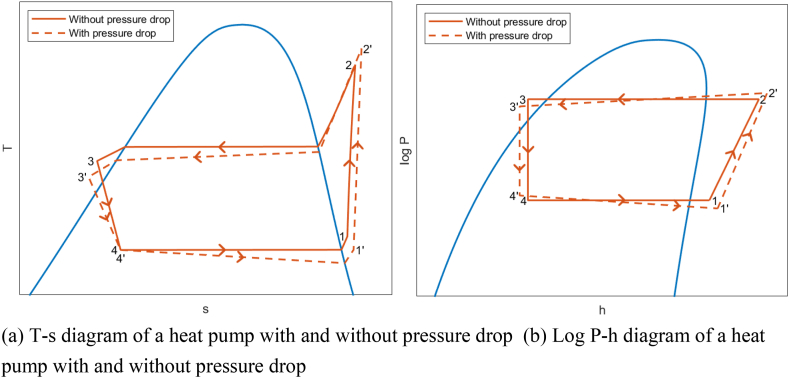
T-s diagram and log P-h diagram of a heat pump with and without pressure drop. (a) T-s diagram of a heat pump with and without pressure drop (b) Log P-h diagram of a heat pump with and without pressure drop.

Many existing models additionally assume isobaric conditions in the evaporator and condenser. However, pressure drop affects the refrigerant properties and therefore also the COP and for that reason this model does take pressure drop into consideration. To illustrate the impact of pressure drop, Fig. 2a displays a T-s diagram, and Fig. 2b presents a log P-h diagram for a heat pump with and without pressure drop.

### 2.2 COP and COP percentage change

The overall aim of this study is to investigate how ambient temperature and relative humidity impact the COP. The COP is defined as:

(1) $$\text{COP}=\frac{\;\text{Heat}\;\text{output}\;}{\;\text{Electricity}\;\text{input}\;}=\frac{{\dot{Q}}_{\text{out}\;}}{{\dot{W}}_{\text{com},\text{el}\;}+{\dot{W}}_{\text{fan},\text{el}\;}}$$

The electrical input to the compressor varies from its work output due to electrical efficiency. Under full load conditions, the compressor is assumed to have an electrical efficiency of 80% [12]. At lower loads, the electrical efficiency is determined using the following equation [12]:

(2) $$\eta_{el}=\left(0.2\;\ln\;\left(\text{LR}\right)+1\right)\eta_{el,fullLoad}$$

This calculation relies on the load ratio (LR), which is defined as:

(3) $$\text{LR}=\frac{{\dot{W}}_{\text{com}\;}}{{\dot{W}}_{\text{com},\text{fullLoad}\;}}$$

To determine how much the COP changes compared to the reference state COP, the COP percentage change is computed:

(4) $${\text{COP}}_{pc}=\left(\frac{{\text{COP}}_{n}}{{\text{COP}}_{r}}-1\right)\cdot 100\%$$

### 2.3 Solving procedure

The simulation model is developed in Matlab. The user first inputs data of a reference heat pump [16] for which the COP is known under EN 14511 standard conditions. The reference heat pump parameters for the simulation model in this study are summarized in Table 1. When pressure drop is disabled in the model, the geometrical parameters of the evaporator and con-denser do not need to be provided.

**Table 1:** Reference heat pump data.

Input parameter	Mitsubishi SUHZ-SW45VA [16]
Ambient temperature	7 °C
Relative humidity	86.8%
Water outlet temperature	35 °C
Water inlet temperature	30 °C
Water pressure	1 bar
Heating capacity (with full load compressor)	7 kW
COP	3.99
Refrigerant	R410A
Refrigerant temperature evaporator inlet	0 °C
Evaporator fan electricity consumption	60 W
Evaporator fan air flow rate	44.6 m^3^/min
Compressor electrical efficiency full load	80%
Compressor isentropic efficiency	80%
Evaporator pipe inside diameter	11.4 mm
Evaporator pipe length	18 m
Condenser corrugation depth	3 mm
Condenser corrugation wavelength	7 mm
Condenser effective plate length	300 mm
Condenser number of plates	20

**Fig. 3:**
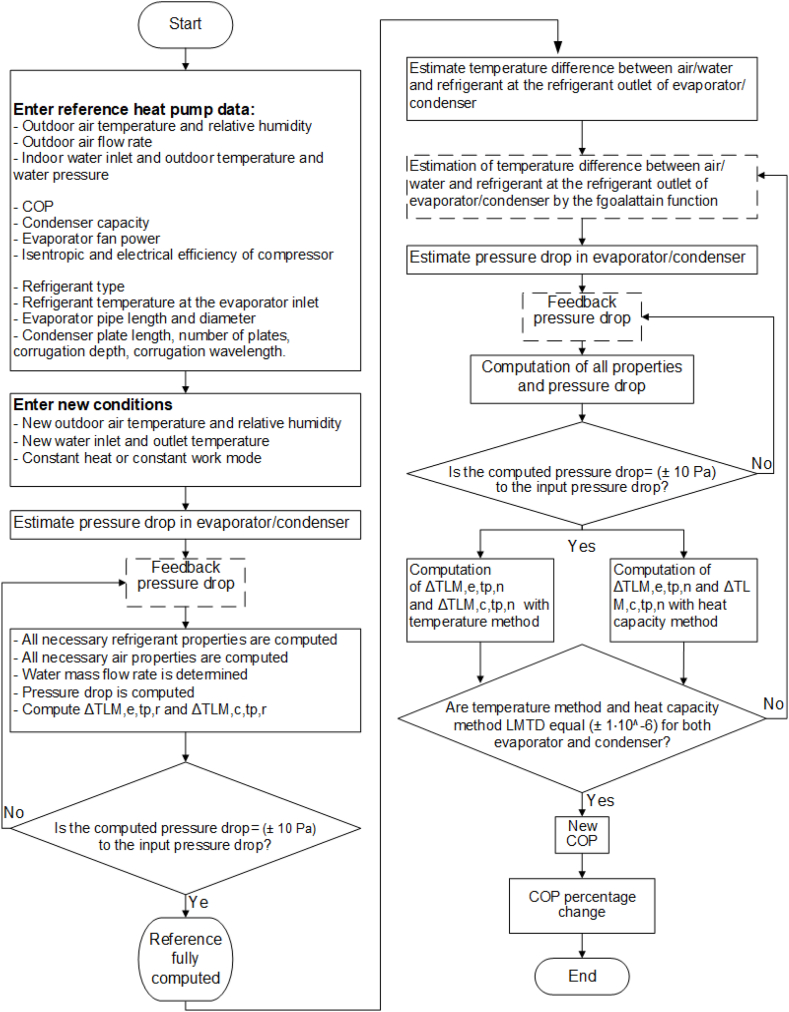
Solving procedure of the simulation model.

In the initial calculation, all unknown parameters under reference conditions are computed, and pressure drop is incorporated using an iterative method. Subsequently, the heat pump model is solved with new environmental conditions, again using an iterative method. Initially, the temperature difference between air/water and refrigerant at the evaporator/condenser refrigerant outlet is guessed. The LMTDs of the evaporator and condenser are calculated with two different methods. If different results arise from these methods, the temperature difference guesses are adjusted using Matlab's ‘fgoalattain’ function until both methods return the same LMTD. The original version of the model, as developed by Sezen & Gungor [12], worked by first guessing the temperature difference at the evaporator until it was accurate, followed by a similar process for the condenser. If the evaporator temperature difference changed after solving for the condenser temperature difference, the process was repeated. The new parallel approach utilizing Matlab's fgoalattain function is significantly more efficient. Finally, the new COP and the COP percentage change are computed. The complete procedure is detailed in Fig. 3. An advantage compared to other simulation methods is that no heat transfer coefficient correlations are required.

### 2.4 Heat pump modes

Two operational modes are available for the heat pump: constant heat mode (CH) and constant work mode (CW). In CH mode, users select their desired condenser heat output. Depending on the ambient conditions, the compressor's work input and evaporator's heat input varies. Under the reference conditions for the reference heat pump [16], the compressor operates at full load. Therefore, achieving the reference heat output of 7 kW is only possible in warmer and/or more humid environmental conditions, where less compressor work is required to generate the same amount of heat. Conversely, in colder and/or less humid conditions, more work input is necessary to achieve the same heat output, which is not feasible because the compressor is already operating at full load. There exists a maximum condenser heat output attainable under specific ambient conditions because the maximum compressor work output cannot be exceeded. In CW mode, users select the compressor's work output. The maximum work output of the compressor cannot be exceeded. In this proposed scenario, we assumed the compressor with constant work condition. The condenser's heat output is then calculated based on the prevailing ambient conditions.

### 2.5 Thermodynamic equations

The model uses thermodynamic equations to analyze the four components of the heat pump. The compressor's work input is calculated using:

(5) $${\dot{W}}_{\text{com}\;}={\dot{m}}_{r}\left(h_{2}-h_{1}\right)=\frac{{\dot{m}}_{r}\left(h_{2,s}-h_{1}\right)}{\eta_{s}}$$

The heat input at the evaporator can be derived either from the refrigerant side or the air side:

(6) $${\dot{Q}}_{e}={\dot{m}}_{r}\left(h_{1}-h_{4}\right)={\dot{m}}_{a}\left(h_{a,e,i}-h_{a,e,o}\right)$$

Similarly, the heat output at the condenser can be determined from either the refrigerant side or the water side:

(7) $${\dot{Q}}_{c}={\dot{m}}_{r}\left(h_{2}-h_{3}\right)={\dot{m}}_{w}\left(h_{w,c,o}-h_{w,c,i}\right)$$

The expansion valve is assumed to be isenthalpic.

(8) $$h_{3}=h_{4}$$

Together with the input parameters provided to the model, the above equations are employed to determine refrigerant, air and water mass flow rates, enthalpies as well as the work or heat input or output of specific components. Additionally, CoolProp [17] is used to determine other properties such as temperature or specific entropy.

### 2.6 Logarithmic mean temperature differences

#### 2.6.1 LMTDs with temperature method

Once all necessary refrigerant, air and water temperatures are determined, the LMTDs in both the evaporator and condenser can be computed. Evaporator and condenser are assumed to be counter-flow heat exchangers.

**Fig. 4:**
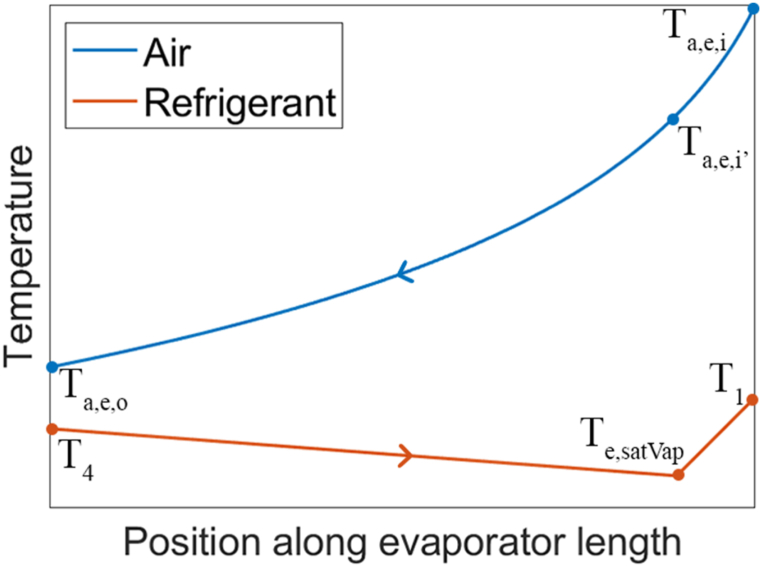
Temperature change of refrigerant and air along the evaporator.

In the evaporator, first the refrigerant is evaporated and then it is superheated by 5 °C. Under isobaric conditions, the temperature remains constant during a phase-change process. How-ever, in this study, pressure drop is considered, which results in temperature changes during the phase-change. Fig. 4 provides a visual representation of how the temperatures of the refrigerant and air change along the length of the evaporator.

Due to the phase change, the overall shape of the refrigerant temperature curve is not logarithmic. Consequently, two distinct LMTDs must be defined: one for the two-phase zone and another for the superheated zone. For the solution procedure, the focus is on the LMTD of the two-phase section, as the majority of the heat transfer occurs in this region. The LMTD for the two-phase section is computed as:

(9) $$\Delta T_{LM,e,tp}=\frac{\left(T_{a,e,i^{\prime }}-T_{e,satVap}\right)-\left(T_{a,e,o}-T_{4}\right)}{\ln\;\left(\frac{T_{a,e,i^{\prime }}-T_{e,satVap}}{T_{a,e,o}-T_{4}}\right)}$$

$T_{e,\text{satVap}\;}$ represents the refrigerant temperature at the saturated vapor state, which is lower than T4 due to pressure drop. $T_{a,e,i^{\prime }}$ is the air temperature at the saturated vapor point of the evaporator. It can be determined using CoolProp [17] and the air enthalpy at that specific point, found using an energy balance with the refrigerant's heat input in the two-phase section:

(10) $${\dot{Q}}_{e,tp}={\dot{m}}_{r}\left(h_{e,satVap}-h_{4}\right)={\dot{m}}_{a}\left(h_{a,e,i^{\prime }}-h_{a,e,o}\right)$$

**Fig. 5:**
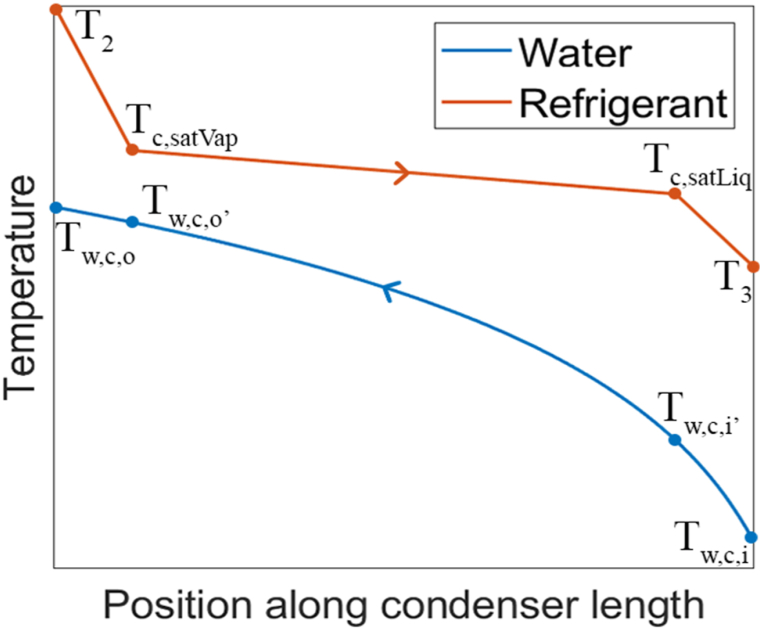
Temperature change of refrigerant and water along the condenser.

In the condenser, first the refrigerant is cooled until it condenses, and after condensation it is 5 °C subcooled. Therefore, three LMTDs can be defined. However, for the solution procedure, the LMTD of the second section where the refrigerant condenses is used, as this section is responsible for the majority of the heat transfer. Fig. 5 illustrates the temperature changes of the refrigerant and water along the length of the condenser.

For the two-phase section in the condenser, the LMTD is determined as follows:

(11) $$\Delta T_{LM,c,tp}=\frac{\left(T_{c,satVap}-T_{w,c,o^{\prime }}\right)-\left(T_{c,satLiq}-T_{w,c,i^{\prime }}\right)}{\ln\;\left(\frac{T_{c,satVap}-T_{w,c,o^{\prime }}}{T_{c,satLiq}-T_{w,c,i^{\prime }}}\right)}$$

$T_{c,\text{satLiq}\;}$ represents the refrigerant temperature at the saturated liquid state, which is lower than the temperature at the saturated vapor state $T_{c,satVap}$ due to pressure drop. $T_{w,c,i^{\prime }}$ corresponds to the water temperature when the refrigerant is in the saturated liquid state and $T_{w,c,o^{\prime }}$ is the water temperature when the refrigerant is in the saturated vapor state. $T_{w,c,i^{\prime }}$ can be determined using CoolProp [17] and the water enthalpy at that specific point, calculated through an energy balance with the refrigerant's heat output in the subcooled section of the condenser:

(12) $${\dot{Q}}_{c,sub}={\dot{m}}_{r}\left(h_{c,satLiq}-h_{3}\right)={\dot{m}}_{w}\left(h_{w,c,i^{\prime }}-h_{w,c,i}\right)$$

Similarly, $T_{w,c,o^{\prime }}$ can be found using CoolProp [17] and the water enthalpy at that point, which can be determined using an energy balance with the refrigerant's heat output in the superheated section of the condenser:

(13) $${\dot{Q}}_{c,\sup}={\dot{m}}_{r}\left(h_{2}-h_{c,satVap}\right)={\dot{m}}_{w}\left(h_{w,c,o}-h_{w,c,o^{\prime }}\right)$$

#### 2.6.2 LMTDs with heat capacity method

$\Delta T_{LM,e,tp}$ and $\Delta T_{LM,c,tp}$ can also be determined for the new ambient conditions using the heat capacity method. The heat transfer provided by heat exchanger can be calculated using the following equation:

(14) $$\dot{Q}=UA_{s}\Delta T_{LM}$$

Assuming that the overall heat transfer coefficient *U* remains constant for both new and reference ambient conditions due to a constant air flow rate, the ratio of heat transfer for the two-phase evaporator section under the new conditions compared to the reference conditions can be established as follows:

(15) $$\frac{{\dot{Q}}_{e,tp,n}}{{\dot{Q}}_{e,tp,r}}=\frac{A_{e,tp,n}}{A_{e,tp,r}}\frac{\Delta T_{LM,e,tp,n}}{\Delta T_{LM,e,tp,r}}$$

Solving for $\Delta T_{LM,e,tp,n}$ and rewriting the equation yields:

(16) $$\Delta T_{LM,e,tp,n}=\Delta T_{LM,e,tp,r}\frac{{\dot{Q}}_{e,tp,n}}{{\dot{Q}}_{e,tp,r}}\frac{\frac{A_{e,tp,r}}{A_{e}}}{\frac{A_{e,tp,n}}{A_{e}}}$$

where:

(17) $$\frac{A_{e,tp}}{A_{e}}=\frac{A_{e,tp}}{A_{e,tp}+A_{e,\sup}}=\frac{\frac{A_{e,tp}}{A_{e,\sup}}}{\frac{A_{e,tp}}{A_{e,\sup}}+1}$$

where:

(18) $$\frac{A_{e,tp}}{A_{e,\sup}}=\frac{{\dot{Q}}_{e,tp}}{{\dot{Q}}_{e,\sup}}\frac{\Delta T_{LM,e,\sup}}{\Delta T_{LM,e,tp}}$$

A similar approach can be applied to the condenser, but with a three-way area split instead of a two-way split:

(19) $$\frac{{\dot{Q}}_{c,tp,n}}{{\dot{Q}}_{c,tp,r}}=\frac{A_{c,tp,n}}{A_{c,tp,r}}\frac{\Delta T_{LM,c,tp,n}}{\Delta T_{LM,c,tp,r}}$$

Solving for $\Delta T_{LM,c,tp,n}$ and rewriting the equation gives:

(20) $$\Delta T_{LM,c,tp,n}=\Delta T_{LM,c,tp,r}\frac{{\dot{Q}}_{c,tp,n}}{{\dot{Q}}_{c,tp,r}}\frac{\frac{A_{c,tp,r}}{A_{c}}}{\frac{A_{c,tp,n}}{A_{c}}}$$

where,

(21) $$\frac{A_{c,tp}}{A_{c}}=\frac{A_{c,tp}}{A_{c,\text{sub}\;}+A_{c,tp}+A_{c,\sup\;}}=\frac{\frac{A_{c,tp}}{A_{c,\text{suup}\;}}}{\frac{A_{c,sub}}{A_{c,\sup}}+\frac{A_{c,tp}}{A_{c,\sup}}+1}$$

where,

(22) $$\frac{A_{c,tp}}{A_{c,\sup\;}}=\frac{{\dot{Q}}_{c,tp}}{{\dot{Q}}_{c,\sup\;}}\frac{\Delta T_{LM,c,\sup\;}}{\Delta T_{LM,c,tp}}$$

and:

(23) $$\frac{A_{c,\text{sub}\;}}{A_{c,\sup\;}}=\frac{{\dot{Q}}_{c,\text{sub}\;}}{{\dot{Q}}_{c,\sup\;}}\frac{\Delta T_{LM,c,\sup\;}}{\Delta T_{LM,c,\text{sub}\;}}$$

### 2.7 Pressure drop

As a novelty compared to other studies, pressure drop in evaporator and condenser is modeled. In real heat pumps, a small amount of oil is added to the refrigerant for lubricating the compressor. In this study, the refrigerant is taken as pure in order to simplify the simulation process. Neglecting the lubricant oil content results in an underestimation of the pressure drop [18,19].

#### 2.7.1 Evaporator pressure drop

The reference heat pump [16] uses a finned-tube heat exchanger as its evaporator. A finned-tube heat exchanger comprises a lengthy, coiled pipe with numerous fins to enhance heat transfer by increasing the available surface area for heat exchange. For simplification purposes, in this model, the finned tube heat exchanger is represented as a horizontal smooth pipe.

In the evaporator, the two-phase refrigerant is first fully evaporated and then superheated 5 °C. In both sections, frictional pressure drop contributes to the overall pressure drop. In the two-phase zone, an additional factor considered is acceleration pressure drop, stemming from the substantial volume change during evaporation, leading to significant acceleration effects. The total pressure drop in the evaporator is computed as follows:

(24) $$\Delta P_{t}=\Delta P_{f,tp}+\Delta P_{acc,tp}+\Delta P_{f,\sup}$$

The Darcy-Weisbach equation [20] is used to calculate frictional pressure drop:

(25) $$\Delta P_{f}=f\frac{L}{D}\frac{v_{m}G^{2}}{2}$$

The mass flux G is defined as the mass flow rate per unit of cross-sectional area:

(26) $$G=\frac{{\dot{m}}_{r}}{A_{c}}$$

The acceleration pressure drop can be described by the following equation [21]:

(27) $$\Delta P_{\text{acc}\;}=\left(v_{\text{out}\;}-v_{\text{in}\;}\right)G^{2}$$

The Darcy friction factor for the two-phase zone *f*_e,tp_ is computed using a homogeneous equilibrium model correlation by Choi et al. [22], specifically developed for refrigerants:

(28) $$f_{e,tp}=0.02024{\text{Re}}_{l}^{-0.0951}\;{\;K}^{0.1554}$$

${\text{Re}}_{l}$ is the liquid-only Reynolds number:

(29) $${\text{Re}}_{l}=\frac{GD}{\mu_{l}}$$

K is the two-phase number:

(30) $$K=\frac{\Delta xh_{lv}}{L_{e,tp}g}$$

In the superheated section of the evaporator, there is single-phase flow. The Darcy friction factor for single-phase flow in a smooth pipe can be computed using the Blasius correlation [23]:

(31) $$f_{e,\sup}=\frac{0.184}{Re_{v}^{0.2}}$$

This form of the Blasius correlation is valid for 2⋅10^4 < Re_v ≤ 2⋅10^6 [23], a range that consistently applies to the modeled heat pump.

#### 2.7.2 Condenser pressure drop

The condenser used in the reference heat pump [16] is a plate heat exchanger. In a plate heat exchanger condenser, various factors contribute to pressure changes, including frictional pressure drop, manifold pressure drop, elevation pressure rise, and deceleration pressure rise [24]. However, this study omits consideration of elevation pressure rise and manifold pressure drop due to their minimal impact on the overall pressure drop. The predominant contributor to the total pressure drop is frictional pressure drop, accounting for 91–99% of the total pressure drop [24]. In the condenser, the refrigerant is first cooled until the saturated vapor state, then it undergoes condensation and finally it is 5 °C subcooled. Frictional pressure drop occurs in all three sections of the condenser. Deceleration pressure rise is only significant in the condensation section, where the refrigerant substantially changes volume due to the phase-change. Consequently, the total pressure drop for the plate heat exchanger in the model is determined as follows:

(32) $$\Delta P_{t}=\Delta P_{f,\sup}+\Delta P_{f,tp}+\Delta P_{\text{dec},tp}+\Delta P_{f,sub}$$

$\Delta P_{\text{dec},tp}$ can be computed using Equation (27). This equation yields a negative value as the outlet volume is smaller than the inlet volume during condensation, resulting in a pressure rise.

The Darcy friction factor for the two-phase zone is calculated utilizing a homogeneous equilibrium model correlation by Zhang et al. [25], explicitly developed for plate heat exchanger condensers for heat pumps:

(33) $$f_{c,tp}=11557.62{\text{Re}}_{eq}^{-1.0041}\;{\text{Bd}}^{0.3002}{\left(\frac{\rho_{l}}{\rho_{v}}\right)}^{-0.4268}$$

${\text{Re}}_{eq}$ is defined as:

(34) $${\text{Re}}_{eq}=\frac{G_{eq}D_{h}}{\mu_{l}}$$

with the equivalent mass flux $G_{eq}$ :

(35) $$G_{eq}=G\left(1-x_{m}+x_{m}{\left(\frac{\rho_{l}}{\rho_{v}}\right)}^{0.5}\right)$$

The mass flux for a PHE is calculated as [26]:

(36) $$G=\frac{{\dot{m}}_{r}}{A_{c}}=\frac{{\dot{m}}_{r}}{\frac{3}{2}\pi D_{h}^{2}}$$

The Bond number Bd is computed as:

(37) $$\text{Bd}=g\left(\rho_{l}-\rho_{v}\right)\frac{D_{h}^{2}}{\sigma }$$

The hydraulic diameter of a PHE is defined as:

(38) $$D_{h}=\frac{2b}{\varphi }$$

φ represents the area enlargement factor:

(39) $$\varphi =\frac{1+\sqrt{1+\gamma^{2}}+4\sqrt{1+\frac{\gamma^{2}}{2}}}{6}$$

γ is a dimensionless corrugation parameter:

(40) $$\gamma =\frac{\pi b}{\lambda }$$

For the subcooled and superheated sections, a single-phase correlation by Jokar et al. is used [26]:

(41) $$f_{c,sub/\;\sup\;}=\frac{25.724}{Re_{l/v}^{0.25}}$$

${\text{Re}}_{l/v}$ is the vapor only Reynolds number for the superheated section (Equation (29)) and the liquid only Reynolds number for the subcooled section. The friction factors of the different condenser sections are then used in Equation (25) to compute the respective frictional pressure drops.

## 3 Results and discussion

### 3.1 Model settings and verification

To analyze the effect of ambient temperature and relative humidity on the COP and the pressure drop, a parallel variation is performed in which either temperature or relative humidity is altered, while keeping the other value constant. Relative humidity is adjusted from 0% to 100%, while ambient temperature is varied from −10 °C to 20 °C, representing typical conditions in most climates. In CH mode, a fixed condenser heat output of 5 kW is used, which is achievable without exceeding the maximum compressor work output even at low ambient temperatures. In CW mode, the compressor work output is set to the maximum possible level. The water inlet temperature is kept at 30 °C, and the water outlet temperature is fixed at 35 °C, reflecting realistic values for modern houses equipped with floor heating.

**Fig. 6:**
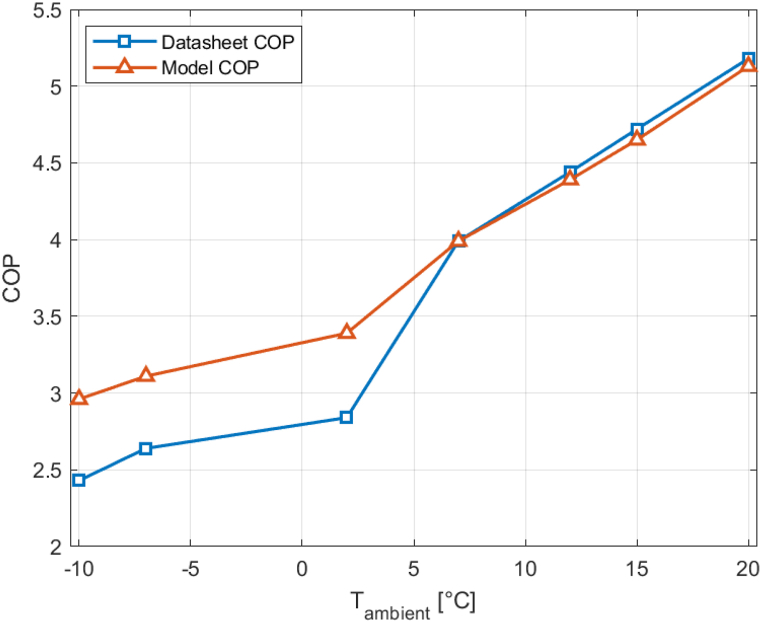
Datasheet and model COP values for a water outlet temperature of 35 ^*◦*^C and a water inlet temperature of 30 ^*◦*^C.

To verify the model's accuracy in simulating a heat pump, seven measured COP values from the reference heat pump datasheet [16] are compared to simulated COP values. Relative humidity varies between different ambient temperatures, as the datasheet conditions according to EN 14511 use a fixed wet bulb temperature that is 1 °C lower than the dry bulb temperature, instead of a fixed relative humidity. RH is computed from the provided wet and dry bulb temperatures and then input into the simulation model along with the ambient temperature. The model is executed, and the simulation model COP is compared to the datasheet COP. Relative humidity is in all cases high enough for frost formation. The comparison results are presented in Fig. 6.

At an ambient temperature of 2 °C and below, the COP is significantly overestimated by the simulation model, with a maximum deviation of 18%. This outcome is expected because at low ambient temperatures coupled with high relative humidity, frost forms on the evaporator. Refrigerant below 0 °C flows through the evaporator, causing its surface temperature to decrease. The already cold and humid external air cools further, going below its dew point due to the low evaporator surface temperature, resulting in the condensation of water vapor. When the evaporator surface temperature drops below 0 °C, the condensate freezes, forming a frost layer on the evaporator. The frost layer increases the thermal resistance of the evaporator, reducing its heat transfer coefficient. Additionally, frost leads to an increase in air pressure drop across the evaporator, leading to reduced airflow and, consequently, decreased heat transfer. To mitigate these two negative effects, heat pumps are typically equipped with a defrosting mode, which can involve cycle reversal, hot gas bypass, or electric resistance heating. However, defrosting comes at a cost to the COP, as electricity is consumed without generating useful heat [27]. Modeling frost formation and defrosting is highly complex, and therefore, a defrosting mode has not been incorporated into the simulation. A potential future research project could be initiated to develop a modeling approach for frost formation and defrosting that can be seamlessly integrated into the existing simulation model. Since the current model does not account for frost formation and defrosting, it overestimates the COP under frosting conditions. At ambient temperatures of 7 °C and above, the model closely aligns with the measured COP values from the datasheet, with only a slight underestimation of 1.5%. The model is evidently suitable for simulations under non-frosting conditions.

### 3.2 Effect of ambient temperature on the condenser capacity and compressor work

**Fig. 7:**
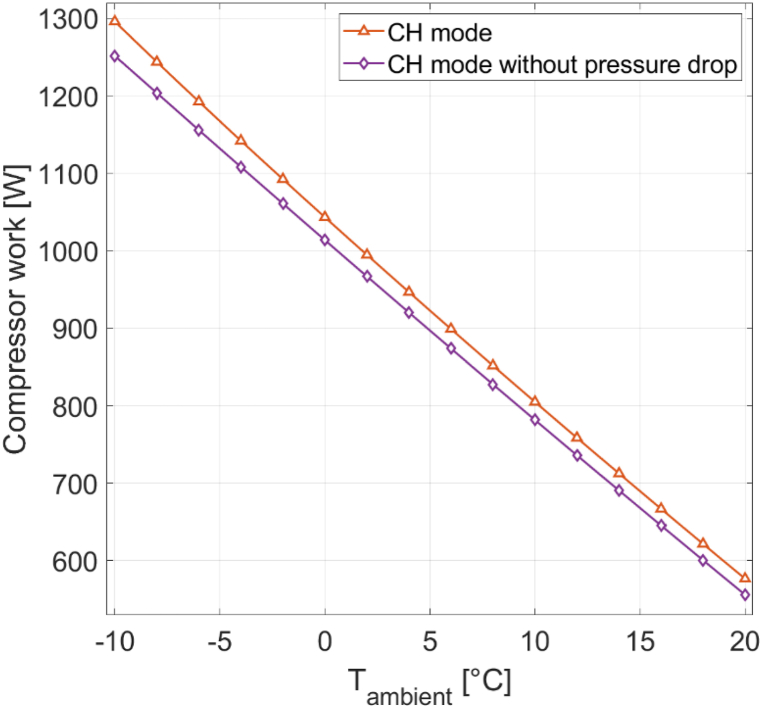
Compressor work for different ambient temperatures with and without pressure drop at 0% RH.

In CH mode, the condenser heat output remains constant while the compressor work output changes. Analyzing the compressor power demand is important, as it is the main contributor to the heat pump's electricity consumption, serving as the denominator in the COP equation (Equation (1)). Fig. 7 shows the compressor work for the reference heat pump [16] under various ambient temperatures, both with and without accounting for pressure drop, under 0% RH.

**Fig. 8:**
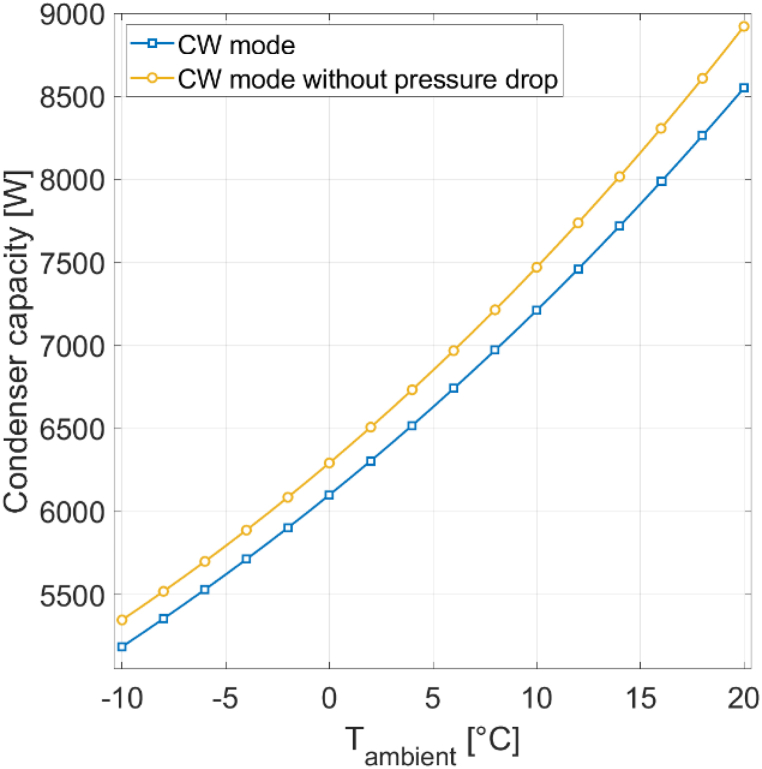
Condenser heat output for different ambient temperatures with and without pressure drop at 0% RH.

At lower ambient temperatures, more compressor work is required to achieve the same amount of heat output. The reason is the increased temperature lift within the heat pump cycle when operating in colder ambient conditions. A higher temperature lift implies the need for a larger pressure differential between the evaporator and condenser sides, which in turn requires increased compressor work. For instance, at −10 °C, 1296 W of compressor work is required to produce 5000 W of heat, whereas at 20 °C, only 577 W is needed. When pressure drop is disabled in the model, the compressor's work demand decreases. At −10 °C, this reduction amounts to 45 W less for achieving a 5000 W condenser heat output. As ambient temperature increases, the impact of pressure drop on compressor work diminishes. At 20 °C, the difference in compressor work with and without pressure drop is only 21 W. In CW mode, the compressor's work is fixed while the condenser heat output varies. Analyzing the condenser's heat output is important, as it serves as the numerator of the COP equation (Equation (1)). Fig. 8 shows the condenser's heat output for the reference heat pump [16], considering both cases with and without pressure drop, under 0% RH conditions.

Lower ambient temperatures lead to a reduction in condenser heat output when compressor work is held constant. For instance, at an ambient temperature of −10 °C, the condenser can deliver 5185 W of heat at full load, whereas at 20 °C, the condenser's capacity increases to 8549 W. Neglecting the influence of pressure drop results in an increase of condenser heat output. At −10 °C, the heat output is increased by 161 W. Disabling pressure drop at higher ambient temperatures has a stronger effect on the heat output than at lower ambient temperatures. In other words, the negative impact of pressure drop is stronger at elevated ambient temperatures. At 20 °C, the condenser's heat output increases by 371 W when pressure drop is disabled in the model.

### 3.3 Effect of ambient temperature and relative humidity on the COP

**Fig. 9:**
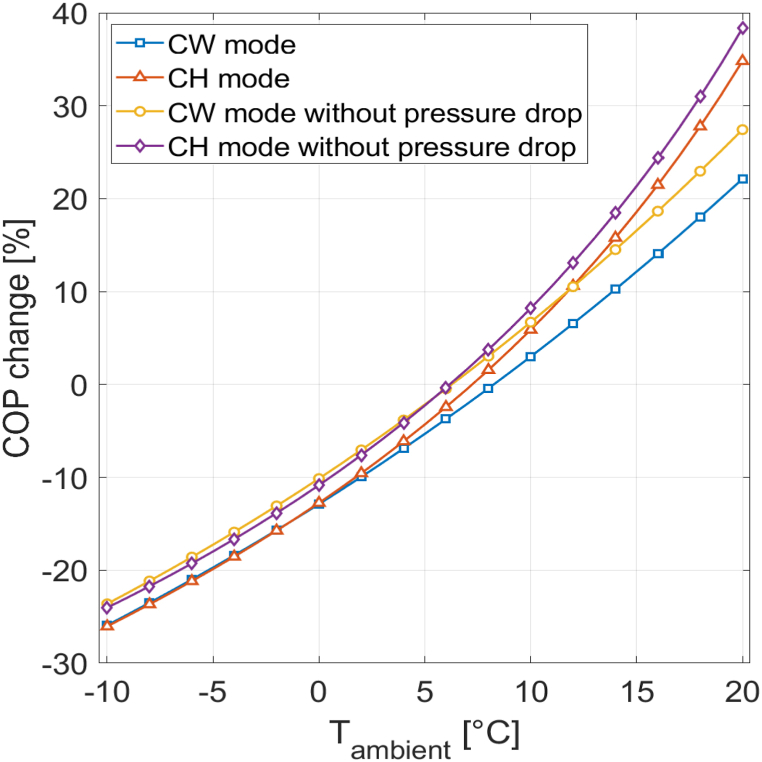
COP percentage change for different ambient temperatures at 0% RH.

Fig. 9 presents the COP percentage change for both CW and CH modes under different ambient temperatures at a relative humidity of 0%, with and without pressure drop. At ambient temperatures exceeding the reference value of 7 °C, a COP improvement is observed, while at lower ambient temperatures the COP decreases. With pressure drop considered, the COP per-centage change at 20 °C compared to 7 °C is 35% for CH mode and 22% for CW mode. At −10.

C, the COP percentage change for CH mode and CW mode is −26%. When pressure drop is not considered, the COP percentage change experiences a slight improvement, increasing by 3.5 percentage points at 20 °C for CH mode and by 5 percentage points for CW mode. The performance difference between enabled and disabled pressure drop is less pronounced at lower ambient temperatures. At −10 °C, the COP percentage change de-crease is 2 percentage points lower without pressure drop for CH mode, and 2.5 percentage points lower for CW mode.

**Fig. 10:**
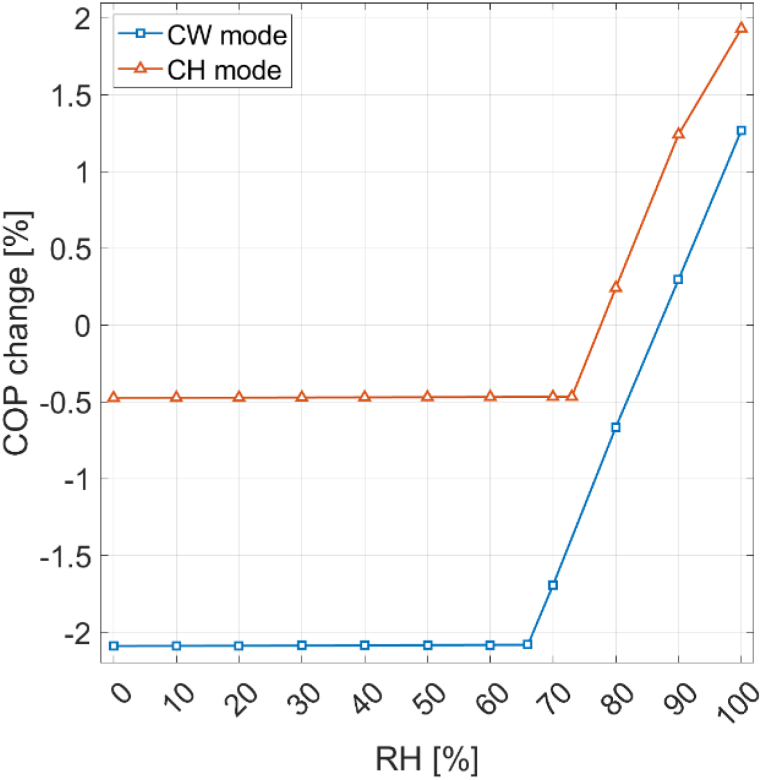
Effect of relative humidity on the COP at an ambient temperature of 7 ^*◦*^C.

The effect of relative humidity on the COP at an ambient temperature of 7 °C for CW and CH mode is pictured in Fig. 10. It becomes apparent that relative humidity does not impact the COP until it reaches a value of 66% for CW mode and 73% for CH mode at 7 °C. These values are the condensation limits. Beyond these limits, the moisture in the air surrounding the evaporator can condense after being cooled by the refrigerant. Condensation slows down the temperature decrease of the air due to the latent heat of condensation, resulting in a higher refrigerant temperature and pressure in the evaporator. This is beneficial for the COP, as it reduces the work required by the compressor. At higher relative humidity percentages, condensation occurs at higher air temperatures, further enhancing the positive effect on the COP. At 100% RH, there is a COP gain of 2.4% in CH mode and a COP gain of 3.3% in CW mode solely due to condensation. In CH mode, the condensation limit is higher and the performance gain of condensation is lower, because the heat pump operates at a reduced capacity of 5 kW, reducing the temperature decrease of the evaporator air. However, condensation can also be detrimental to performance at low ambient temperatures. A low ambient temperature combined with high RH leads to frost formation on the evaporator surface due to freezing of air moisture condensate, as discussed in section 3.1. In CW mode at 100% RH, frosting occurs at 3.1 °C ambient temperature and below. At lower relative humidities above the condensation limit, frosting occurs already at higher temperatures, as the air temperature drop is steeper due to delayed condensation. At 70% RH, the frosting limit is 5.3 °C.

### 3.4 Pressure drop

**Fig. 11:**
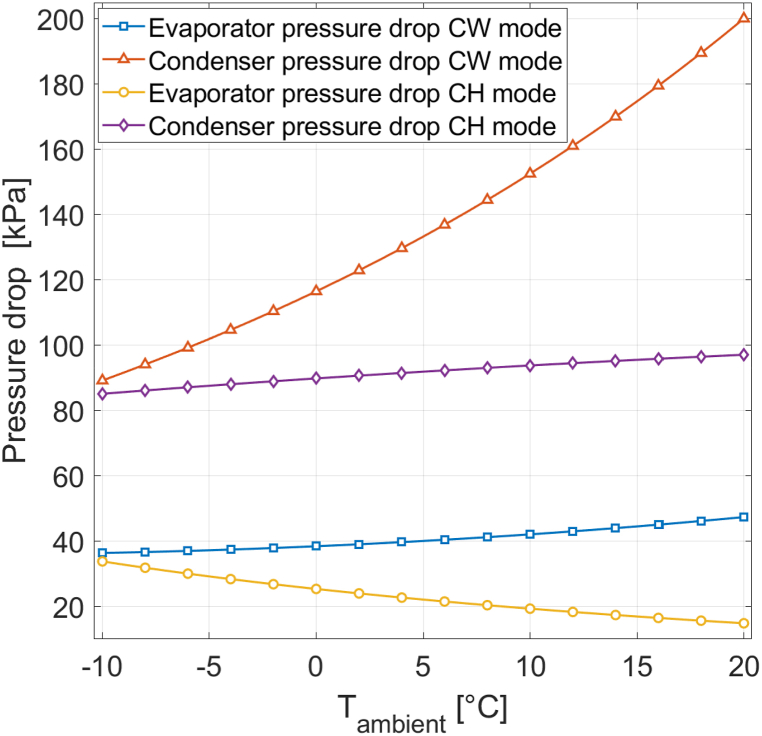
Evaporator and condenser pressure drops for different ambient temperatures at 0% RH.

Fig. 11 presents the evaporator and condenser pressure drops for both CH mode and CW mode at 0% RH. Pressure drop is more pronounced in the condenser than in the evaporator, and is not constant. Pressure drop is mainly affected by the refrigerant mass flow rate. The Darcy- Weisbach frictional pressure drop (Equation (25)) and the acceleration pressure drop (Equation (27)) involve G^2^, the mass flux squared, which scales linearly with the mass flow rate (Equation (26)). The mass flow rate changes with changing ambient conditions. Moreover, refrigerant pressure levels change in response to varying environmental conditions, further impacting pressure drop. The mean refrigerant specific volume $v_{m}$ has a linear influence on frictional pressure drop and is strongly affected by refrigerant pressure.

For the reference heat pump [16], the maximum pressure drops in the condenser and evaporator at 0% RH are 200 kPa and 47 kPa, respectively. Condenser pressure drop is higher in CW mode than in CH mode. This disparity is primarily attributable to the mass flow rate, which is larger in CW mode than in CH mode. Additionally, the rate of change in pressure drop is more significant in CW m ode, mainly due to the substantial fluctuations in mass flow rat e. In the evaporator, pressure drop exhibits only a slight increase at higher environmental temperatures in CW mode and even decreases in CH mode for the reference heat pump [16]. The main reason is the substantial reduction in mean refrigerant volume at higher ambient temperatures due to increased evaporator pressure. This reduction counteracts the pressure drop increase stemming from increased mass flow rate. In CH mode, the effect of the mean volume decrease outweighs the mass flow rate increase, resulting in lower pressure drop at higher ambient temperatures. It is essential to note that for heat pumps other than the reference heat pump [16], the relative impacts of the mass flow rate increase and the mean refrigerant specific volume decrease could differ, making the decrease in evaporator pressure drop at higher ambient temperatures not universally applicable to all heat pumps. Nevertheless, a consistent finding is that the mean refrigerant specific volume experiences more substantial changes in the evaporator than in the condenser because evaporator pressure is more significantly affected by ambient temperature than condenser pressure. Additionally, the mean refrigerant specific volume is more sensitive at lower pressures than at higher pressures. In other words, a 200 kPa pressure change at lower pressures impacts the mean refrigerant specific volume more significantly than a 200 kPa pressure change at higher pressures.

**Fig. 12:**
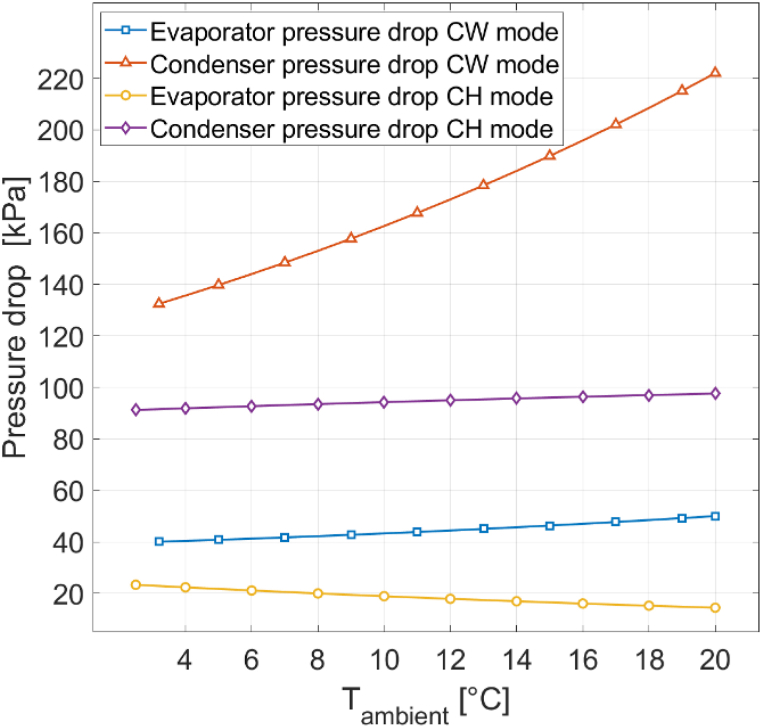
Evaporator and condenser pressure drops for different ambient temperatures at 100% RH.

Fig. 12 displays the evaporator and condenser pressure drops for 100% RH. Results can-not be generated for ambient temperatures below 2.5 °C for CH mode and 3.2 °C for CW mode, due to frosting occurring below these temperatures. The general pressure drop behavior in response to varying ambient temperatures remains consistent. The most notable difference is the 22 kPa increase in condenser pressure drop at 20 °C for CW mode, primarily due to a higher mass flow rate at 100% RH. The 222 kPa condenser pressure drop at 20 °C and 100% RH for CW mode is the overall largest pressure drop for the reference heat pump [16]. The maximum evaporator pressure drop is 50 kPa.

### 3.5 Effect of different condenser capacities

**Fig. 13:**
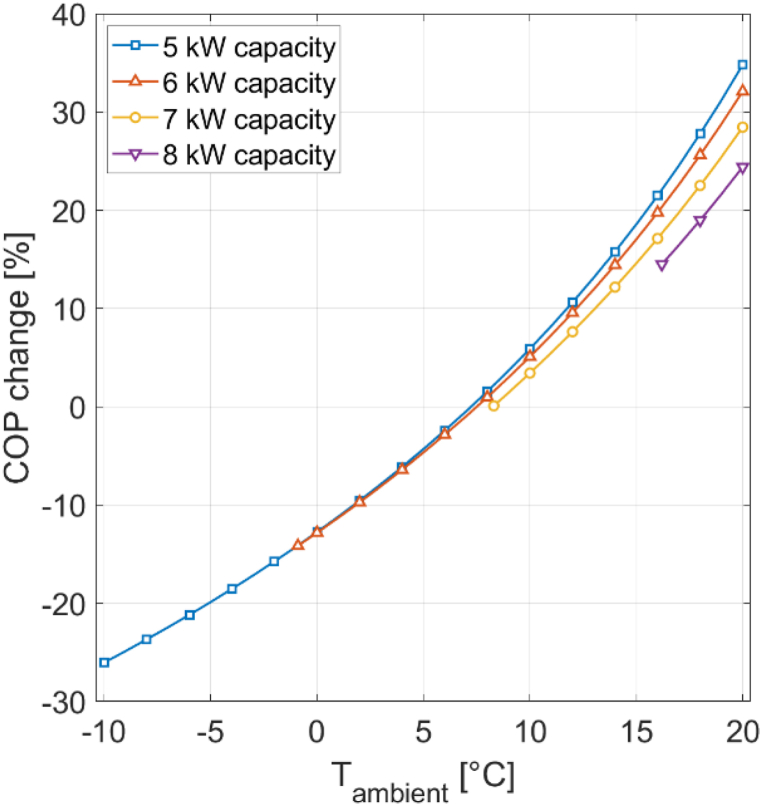
COP percentage change for different ambient temperatures at 0% RH with different condenser capacities.

Previously, the simulation was executed with 5 kW capacity for CH mode. Changing the condenser capacity affects the COP and pressure drop. Fig. 13 shows the impact of varying condenser capacities (5 kW, 6 kW, 7 kW, and 8 kW) on the COP percentage change under different ambient temperatures at 0% RH in CH mode.

Higher condenser capacities and lower ambient temperatures require greater compressor work. As condenser capacity increases, the lowest temperature on the plot increases since the com-pressor cannot be overloaded. It can be seen that higher condenser capacities exert a negative effect on the COP, with a more pronounced effect at higher ambient temperatures. Specifically, at an ambient temperature of 20 °C and a 5 kW condenser capacity a 35% COP gain is achieved compared to the reference state at 7 °C. With an 8 kW condenser capacity, the COP percentage change drops to 24%. With a 6 kW condenser capacity, the COP increase at 20 °C is 32%, showing a 3 percentage point difference compared to a 5 kW condenser capacity. At 0 °C, the distinction between 5 kW and 6 kW condenser capacity is negligible, measuring less than 0.1 percentage points.

**Fig. 14:**
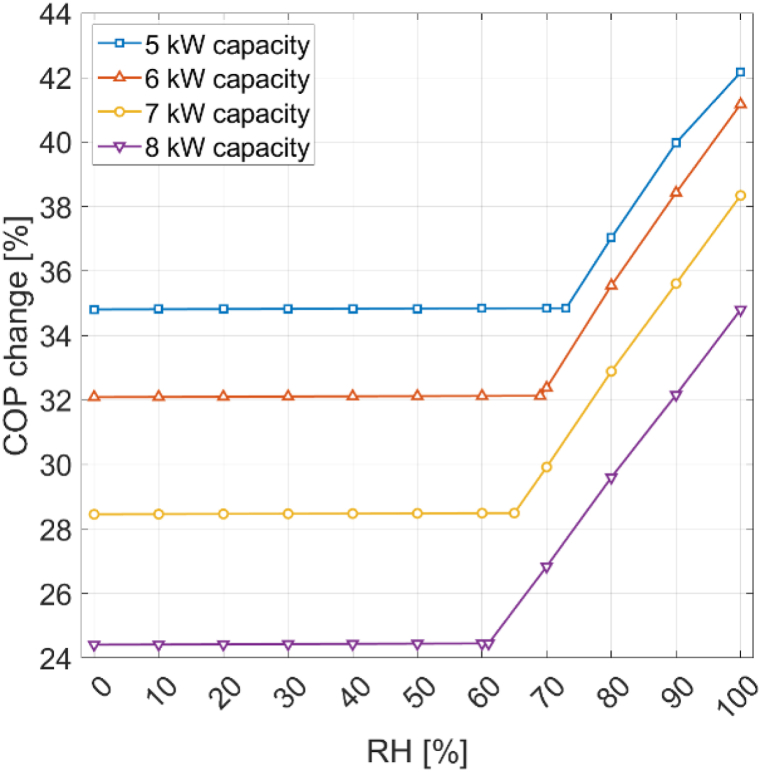
COP percentage change for different relative humidities at 20 °C with different con-denser capacities.

Fig. 14 displays the COP percentage change under different relative humidities at 20 °C for condenser capacities of 5 kW, 6 kW, 7 kW, and 8 kW. Lower condenser capacities have a positive impact on the COP. At 100% RH, the COP percentage change compared to the reference state at 7 °C is 42% for a 5 kW condenser capacity and 35% for an 8 kW capacity. The rate of change is more rapid for larger condenser capacities. The difference between a 5 kW capacity and a 6 kW capacity at 20 °C is only 1 percentage point, while the difference between a 7 kW and an 8 kW capacity is 3.5 percentage points. Additionally, higher condenser capacities lead to lower condensation limits. For a 5 kW capacity, the condensation limit is 73%, whereas for an 8 kW capacity it drops to 61%. The reason is that a greater condenser capacity results in higher evaporator heat input, leading to a more substantial drop in ambient air temperature. The increased air temperature decrease means the dew point is reached even at lower relative humidities, leading to condensation. The lower condensation limit enhances the performance gain from condensation. The COP percentage change at 100% RH compared to no condensation is 10.4% for an 8 kW condenser capacity, but only 7.3% for a 5 kW condenser capacity. However, a lower condensation limit also means that frosting can occur at lower relative humidities when the ambient temperature is sufficiently low. Furthermore, the increased air temperature decrease resulting from larger condenser capacities leads to frosting at higher ambient temperatures.

**Fig. 15:**
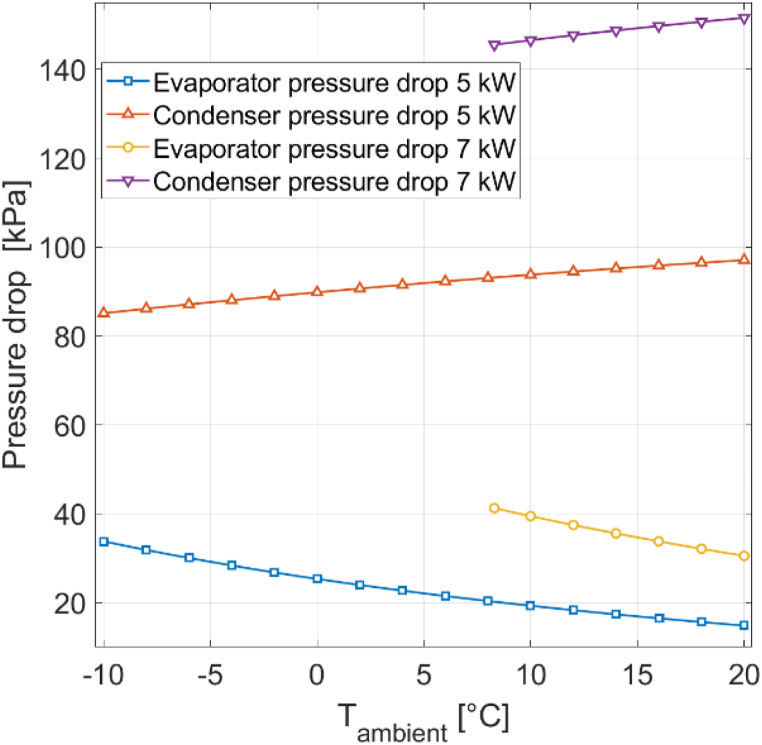
Evaporator and condenser pressure drops at different ambient temperatures at 0% RH for 5 kW and 7 kW condenser capacity.

Fig. 15 presents the evaporator and condenser pressure drops at different ambient temperatures at 0% RH for condenser capacities of 5 kW and 7 kW. For a 7 kW condenser capacity, it is not possible to generate results for ambient temperatures below 8.3 °C, due to compressor overload. Pressure drop is more significant for larger condenser capacities, with the condenser showing a more pronounced difference between different capacities compared to the evaporator. At 20 °C, the condenser pressure drop measures 97 kPa for a 5 kW condenser capacity and 152 kPa for a 7 kW condenser capacity. The evaporator pressure drop at 20 °C is 15 kPa for a 5 kW condenser capacity and 30 kPa for a 7 kW condenser capacity. The pressure drop difference be-tween different capacities is relatively consistent regardless of ambient temperature. Increasing condenser capacity requires a higher refrigerant mass flow rate to ensure adequate heat output in the condenser. An increased mass flow rate elevates the mass flux (Equation (26)), which, in turn, exerts a quadratic influence on frictional and acceleration pressure drop (Equations (25), (27)), leading to larger pressure drops for greater condenser capacities.

### 3.6 Effect on the pressure drop when using different refrigerants

The reference heat pump [16] uses the HFC blend R410A as its refrigerant, which has been a common choice for heat pumps. Over 80% of heat pumps sold in 2019 contained R410A [28]. While R410A does not have ozone depletion potential (ODP) [6], it does pose an environmental challenge due to its high global warming potential (GWP) of 2088 [5]. Consequently, efforts to phase out R410A are underway, particularly in the European Union [5]. An alternative to R410A is the HFC R32, which has a lower GWP of 677 and no ODP [6]. This positions it as a viable refrigerant choice, as it can still be used in single split air-conditioning systems beyond 2025 [5], unlike R410A. In this study, R32 is considered as an alternative refrigerant for comparison, given its potential as a more environmentally friendly direct substitute for R410A [6]. Another promising alternative is R290 (propane), which carries an exceptionally low GWP of 20 [6] and has no ODP [6]. R290 is gaining popularity as a replacement for R410A in various applications [28], although it does have the drawback of flammability [6]. Table 2 summarizes the safety and environmental properties of R410A, R32, and R290 [6]. To assess the impact of switching to different refrigerants on heat pump pressure drop, simulations are executed using R32 and R290 in place of R410A.

**Table 2:** Safety and environmental properties of R410A, R32 and R290.

Refrigerant	Safety Group^1^	ODP	GWP
R410A	A1	0	2088
R32	A2L	0	677
R290	A3	0	20

Significant disparities in condenser pressure drop are observed among the three refrigerants. The highest pressure drop is exhibited by R410A, ranging from 85 to 97 kPa. In contrast, a lower and more constant pressure drop in the range of 70–72 kPa is observed with R290, while the lowest condenser pressure drop is recorded with R32, measuring between 54 and 64 kPa.

Differences in evaporator pressure drop among the three refrigerants are less pronounced. Here, the highest pressure drop is found with R290, ranging from 17 to 39 kPa. R410A follows closely behind, with pressure drop values between 15 and 34 kPa. The lowest evaporator pressure drop is again found with R32, ranging from 10 to 22 kPa. Additionally, as the ambient temperature rises, the pressure drop gap between the three refrigerants diminishes for the evaporator.

**Fig. 16:**
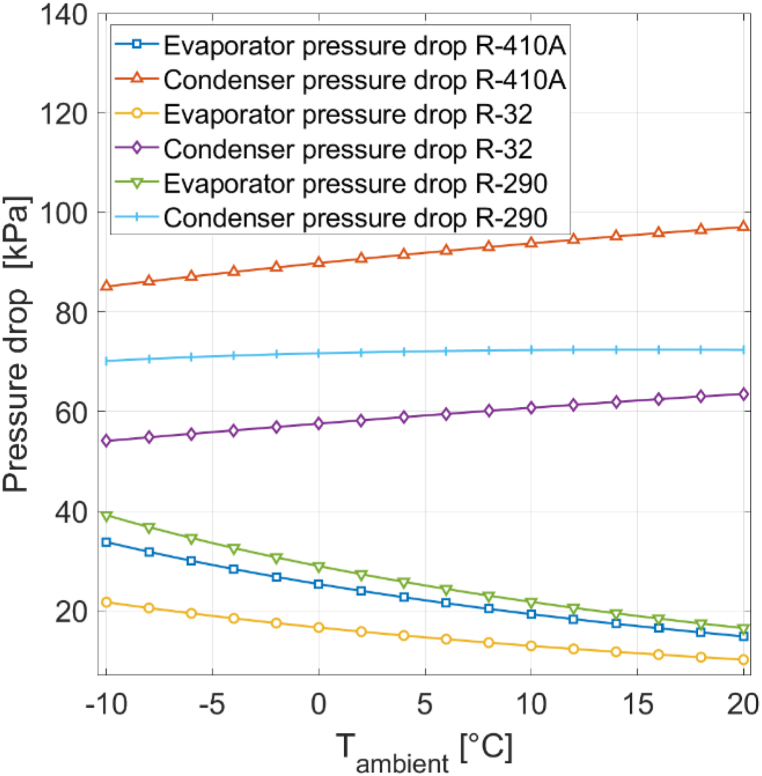
Evaporator and condenser pressure drops for different refrigerants with CH mode at 0% RH.

When comparing condenser and evaporator pressure drop, it is notable that R410A is the refrigerant with the highest condenser pressure drop, but R290 is the refrigerant with the highest evaporator pressure drop. R32 has the lowest pressure drop for both components. Fig. 16 displays the condenser and evaporator pressure drops for CH mode with the three refrigerants under different ambient temperatures at 0% RH.

**Fig. 17:**
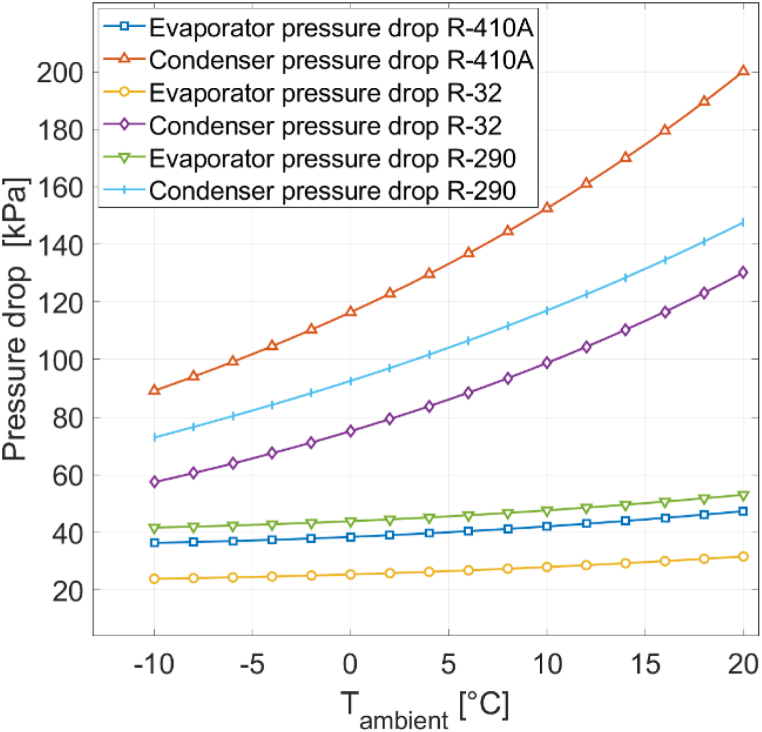
Evaporator and condenser pressure drops for different refrigerants with CW mode at 0% RH.

In Fig. 17, the pressure drops for the three refrigerants under different ambient temperatures at 0% RH in CW mode are presented. Again, differences in pressure drop among the three refrigerants are more pronounced for the condenser than for the evaporator. R410A exhibits the highest condenser pressure drop, varying from 89 to 200 kPa. R290, in contrast, registers a lower pressure drop, fluctuating between 74 and 148 kPa, while R32 maintains the lowest condenser pressure drop, measuring between 57 and 130 kPa.

For the evaporator, the pressure drop disparities between the three refrigerants are smaller. R290 yields the highest evaporator pressure drop, ranging from 42 to 53 kPa. R410A follows closely behind, with pressure drop values between 36 and 47 kPa. R32 maintains the lowest evaporator pressure drop, ranging from 24 to 32 kPa. The results for CW mode align with those for CH mode. R410A consistently shows the highest condenser pressure drop, while R290 consistently yields the highest evaporator pressure drop. In both modes, R32 consistently records the lowest pressure drop. The differences in pressure drop among the three refrigerants are more consistent in CW mode than in CH mode. In CH mode, a higher ambient temperature leads to a reduced pressure drop gap between the refrigerants for the evaporator. Multiple factors contribute to the variations in pressure drop among the three refrigerants. Frictional pressure drop is computed using the Darcy-Weisbach pressure drop formula (Equation (25). Parameters specific to the refrigerant include the mass flux G, the mean specific volume vm, and the Darcy friction factor f. While R290 displays the lowest mass flux and friction factor, its mean specific volume i s significantly larger than that of the other two refrigerants, resulting in a non-minimal pressure drop for R290. On the other hand, R410A has the lowest mean specific volume but features higher mass flux and friction factor than R2 90. R32 does not possess the smallest mass flux, mean specific volume, or friction factor; however, all three relevant parameters are close to the minimum value, and therefore R32 is the refrigerant with the lowest pressure drop.

## 4 Conclusion

A simulation model has been developed to accurately determine the coefficient of performance (COP) of an air-to-water heat pump under non-frosting conditions. The model considers evaporator and condenser pressure drop and relies on the equalization of logarithmic mean temperature differences (LMTDs), computed using two different methods for both the evaporator and condenser. The pressure drop is computed iteratively using pressure drop correlations de-signed for finned-tube heat exchangers and plate heat exchangers. The following key findings can be drawn from this study:

• The proposed model is highly accurate above frosting conditions, with only a marginal deviation of 1.5% compared to COP values from the datasheet.
• Ambient temperature has a strong influence on the COP. A rise in ambient temperature to 20 °C yields a COP increase of up to 35% when compared to an ambient temperature of 7 °C for the reference heat pump [16]. Conversely, an ambient temperature of −10 °C leads to a 26% decrease in COP.
• Relative humidity becomes a performance enhancer once condensation begins. Although the effect of relative humidity on the COP is less pronounced than the impact of ambient temperature, it can still result in performance gains of up to 10.4% for the reference heat pump [16].
• Pressure drop varies based on ambient temperature and relative humidity, with the con-denser displaying larger pressure drop values compared to the evaporator for the reference heat pump [16]. Maximum pressure drops are 220 kPa for the condenser and 50 kPa for the evaporator. Pressure drop negatively affects COP, and disabling pressure drop in the model enhances the COP by up to 5% for the reference heat pump [16].
• Higher condenser capacities decrease the COP, especially at higher ambient temperatures. At 20 °C, a condenser capacity of 8 kW results in a 3% lower COP than a 5 kW condenser capacity. Higher condenser capacities decrease the condensation limit. At 20 °C, the condensation limit is 73% relative humidity with a 5 kW condenser capacity and 61% relative humidity with an 8 kW condenser capacity for the reference heat pump [16].
• Pressure drop characteristics vary with the choice of refrigerant. For the reference heat pump [16], the lowest evaporator and condenser pressure drops are achieved with R32. R410A shows the highest condenser pressure drop, while using R290 results in the maximum evaporator pressure drop.
• The proposed model is designed for predicting the performance of air-to-water heat pumps, incorporating considerations for pressure drops in the evaporator and condenser. To utilize this model for predictions, initial assumption values are required. However, through multiple rounds of iterative loop calculations, the model refines these assumptions, ultimately yielding accurate performance values.

Future research could extend the model with a frosting simulation and a defrosting mode for simulating a heat pump under frosting conditions. Additionally, an extended model could intro-duce a variable heating mode that considers a house's heating requirements based on ambient temperature and relative humidity, dynamically adjusting the condenser capacity accordingly. To further enhance the model, assumptions such as the absence of unwanted heat losses could be eliminated, and a more realistic evaporator model could be developed, accounting for factors like pipe configurations. Moreover, modeling of pressure drop in interconnecting pipes among heat pump components or employing correlations that consider lubricant oil effects could be explored.

## CRediT authorship contribution statement

**Tim Koopman:** Writing – review & editing, Writing – original draft, Validation, Resources, Methodology, Investigation. **Tingting Zhu:** Writing – review & editing, Writing – original draft, Supervision, Methodology, Investigation, Conceptualization. **Wilko Rohlfs:** Writing – review & editing, Supervision, Methodology.

## Declaration of competing interest

We declare that we have no financial and personal relationships with other people or organizations that can inappropriately influence our work, there is no professional or other personal interest of any nature or kind in any product, service and/or company that could be construed as influencing the position presented in, or the review of, the manuscript entitled.

We understand that the Corresponding Author is the sole contact for the Editorial process (including Editorial Manager and direct communications with the office). He is responsible for communicating with the other authors about progress, submissions of revisions and final approval of proofs. We confirm that we have provided a current, correct email address, which is accessible by the Corresponding Author.
